# Neuroprotective effects of *Paeonia Lactiflora* extract against cell death of dopaminergic SH-SY5Y cells is mediated by epigenetic modulation

**DOI:** 10.1186/s12906-016-1205-y

**Published:** 2016-07-12

**Authors:** Gyuhwi Lee, Jong Cheon Joo, Bo Yoon Choi, Anders M. Lindroth, Soo Jung Park, Yoon Jung Park

**Affiliations:** Department of Nutritional Science and Food Management, College of Science and Industry Convergence, Ewha Womans University, Seodaemoon-gu, Seoul 03760 Republic of Korea; Department of Sasang Constitutional Medicine, College of Korean Medicine, Wonkwang University, Iksan, 54538 Republic of Korea; Department of System Cancer Science, Graduate School of Cancer Science and Policy, National Cancer Center, Ilsandong-gu, Goyang, 10408 Republic of Korea; Department of Sasang Constitutional Medicine, College of Korean Medicine, Sangji University, Wonju, 26339 Republic of Korea

**Keywords:** Paeonia lactiflora, Histone acetylation, Epigenetics, MPP^+^-induced cell death, Neuroprotective effect

## Abstract

**Background:**

The *Paeonia lactiflora* extract (PLE) has been reported to have neuroprotective effect against neurodegeneration that are induced by cellular stress such as oxidative stress. Its underlying mechanisms, however, remain unclear. In latest decades, emerging evidence has suggested that epigenetic mechanisms play a key role in gene regulation in response to the cellular stress. We investigated whether epigenetic modulation was involved in neuronal cell death by the neurotoxicant, 1-Methyl-4-phenylpyridinium (MPP^+^), and the neuroprotective effect of PLE.

**Methods:**

Differentiated SH-SY5Y, which is a well-established dopaminergic cell line model, was treated with 0 ~ 200 μg/ml PLE for 4 h prior to MPP^+^ treatment. The effect of PLE on cell viability was determined by MTT assays. Gene expression levels of oxidative stress responsive genes, such as *Heme oxygenase 1* (*HMOX1*), and histone modifiers, such as histone acetyltransferases (HATs) and deacetylases (HDACs) were measured by quantitative RT PCR. In order to investigate the changes in epigenetic modifications, the acetylated lysine 9 (H3K9ac) and lysine 27 (H3K27ac) of Histone H3 were measured by western blot using histones extracted from the cells.

**Results:**

MPP^+^-induced cell death in SH-SY5Y cells was significantly reduced by PLE pretreatment in a dose-dependent manner, indicating the potent neuroprotective effects of PLE. It was accompanied by induced expression of *HMOX1*. MPP^+^ treatment increased the expression of HATs and consistently increased H3K9ac and H3K27ac of Histone H3. PLE pretreatment impeded the changes in H3K9ac and H3K27ac, coincided with increased expression of HDAC5 without changes in HAT expression.

**Conclusions:**

The results suggested that MPP^+^-induced cell death in the dopaminergic SH-SY5Y cells was related with transcriptional induction of HATs and increased histone H3 acetylation and that PLE might prevent the cells from MPP^+^-induced cell death via tempering histone H3 acetylation.

## Background

Parkinson’s disease, the second most common neurodegenerative disorder following Alzheimer’s disease, is mainly caused by progressive degeneration of dopaminergic neurons [1]. One major risk factor leading to the degeneration is cellular oxidative stress [2]. Oxidative stress is a cellular oxidative imbalanced condition due to impaired mitochondrial function and elevated reactive oxygen species (ROS) in the cell. 1-methyl-4-phenyl-1,2,3,6-tetrahydropyridine (MPTP) and its metabolite, MPP^+^, has been used as oxidative stressors that induce cell death in dopaminergic neurons in vivo and in vitro, which mimic Parkinson’s disease [2]. MPP^+^ enters dopaminergic neuronal cells via dopamine transporters and induces mitochondria dysfunction. It produces ROS [3] and releases cytochrome C (Cyt-C), which activates caspase-3 and eventually, leads dopaminergic neuronal cells to apoptosis [4]. MPP^+^-induced cell system serves as a useful model to study the molecular mechanism underlying loss of dopaminergic neurons and therapeutic potentials.

*Paeonia lactiflora* Pall is one of the most commonly used medicinal herbs across different traditional Chinese medicine treatment for its anti-inflammatory and antioxidant effects [5]. The herbal extract of this plant, PLE, has been reported to have neuroprotective effects against oxidative stress both in vivo [6] and in vitro [7, 8], in addition to the main compounds such as albiflorin [9], paeoniflorin [10–12], and paeonol [13, 14] that have been extracted from this plant. It indicates its potentials to protect dopaminergic neurons from cell death and to attenuate the process of Parkinson’s disease [6, 15, 16]. The underlying molecular mechanism of PLE, however, remains elusive.

Recently, the emerging evidence on disease-preventive and therapeutic effects of phytochemicals and herb extracts suggests that they mainly exert their functions, at least in part, via epigenetic modulation [17]. Epigenetic mechanisms such as DNA methylation and histone modifications regulate gene expression via changes in chromatin accessibility in response to environmental stimuli such as oxidative stress [18]. Disturbed patterns of histone acetylation as well as altered activity of HATs and HDACs have been connected to chronic diseases, including cancer and neurodegenerative diseases [18]. Cellular stress alters histone acetylation at specific residues such as histone H3 lysine9 and histone H4 lysine12 [19–21] and changes the activities of HDACs [22, 23], suggesting the potential development of therapeutic strategies for neurodegenerative conditions [24–27]. However, the epigenetic mechanism has been investigated on limited phytochemicals, for examples, curcumin enriched in turmeric and sulforaphane enriched in broccoli, which have effects on cell death and proliferation by inhibiting HATs and HDACs, respectively [28–30]. Consequently, to identify potential modulators of histone acetylation in neuronal cells may provide novel therapeutic avenues in neurodegenerative disorders.

Therefore, we investigated whether the neuroprotective effects of PLE against oxidative stress in dopaminergic neuron cells are mediated by epigenetic modulation. Using the MPP^+^-induced SY5Y cell model, gene expression involving the antioxidant pathway as well as histone acetylation histone acetylation levels at histone H3 lysine 9 (H3K9) and lysine 27 (H3K27) were analyzed with and without PLE treatment.

## Methods

### Cell culture

SH-SY5Y cell line was obtained from the Division of Epigenomics and Cancer Risk Factors, German Cancer Research Cancer (DKFZ, Heidelberg, Germany). The cells were cultured in Dulbecco’s modified Eagle’s medium (DMEM) (Welgene, Daegu, South Korea) supplemented with 10 % fetal bovine serum (FBS) (Gibco, Gaithersberg, MD, USA), 100 units/ml penicillin, and 100 μg/ml streptomycin (Gibco, Gaithersberg, MD, USA) with incubation at 37 °C in humidified 5 % CO_2_ and 95 % air. Upon cell passage, treatment of cells with 0.25 % trypsin-EDTA (Gibco, Gaithersberg, MD, USA), was followed by quenching in DMEM whereupon the cells were collected by centrifugation and rinsed with phosphate buffered saline (PBS) (Sigma Aldrich, St. Louis, MO, USA). The number of viable cells was counted in a hemocytometer under a microscope after excluding dead cells dyed with 0.4 % trypan blue solution (Gibco, Gaithersberg, MD, USA).

### Cell differentiation and treatment

To obtain dopaminergic neuronal cells, SY5Y cells were differentiated using DMEM with 10 μM retinoic acid (Sigma Aldrich, St. Louis, MO, USA), 1 % FBS and100 units/ml penicillin, and 100 μg/ml streptomycin for 4 days. The medium was changed after 2 days during differentiation. The differentiation was confirmed by the increase in mRNA level of *Tyrosine hydroxylase* gene (TH). Differentiated cells were treated with 0-2 mM MPP^+^ (Sigma Aldrich, St. Louis, MO, USA) for 24 h to optimize the concentration of MPP^+^ treatment. To investigate PLE effects, the differentiated cells were pretreated with 0-200 μg/ml PLE for 4 h before 1 mM MPP^+^ treatment. Lyophilized PLE extract was prepared by Hanpoong Pham & Foods Co., Ltd (Jeonju, South Korea). Briefly, 300 g of *Paeonia lactiflora* was refluxed for 3 h in three liters of 30 % ethanol, passed through 0.1 μm filter and vaporized. PLE, obtained with 201.3 % yield, was dissolved in dimethyl sulfoxide (DMSO) (Sigma Aldrich, St. Louis, MO, USA) in 80 mg/ml PLE as a stock solution. The control groups were supplemented with the same amount of DMSO in the final maximum concentration less than 0.25 %.

### MTT assays

3-[4,5-dimethylthiazol-2-yl]-2,5-diphenyltetrazoliumbromide (MTT) (Sigma Aldrich, St. Louis, MO, USA) was dissolved in PBS at the concentration 5 mg/ml. The MTT solution was added to one-tenth the original culture volume and the cells were incubated at 37 °C for 2 h. Then, DMSO was added in cells to convert soluble MTT to insoluble purple formazan in mitochondria of living cells. Viable cells forming formazan were measured at 562 nm using a microplate reader (Lab tech international, Uckfield, UK).

### RNA isolation and reverse transcription

Total RNA was isolated from cells by Trizol reagent (Tel-test Inc, Friendswood, TX, USA). Reverse transcription of 500 ng or 1 μg total RNA was performed for cDNA synthesis. In brief, RNA samples were treated with DNase I (Thermo Fisher Scientific, Waltham, MA, USA) to remove genomic DNA, after which RNA samples were incubated with dNTP (Thermo Fisher Scientific, Waltham, MA, USA), oligo dT (Thermo Fisher Scientific, Waltham, MA, USA), and reverse transcriptase (Thermo Fisher Scientific, Waltham, MA, USA) to synthesize cDNA, following the manufacturer’s protocol.

### Quantitative reverse transcriptase (RT)-PCR

Quantitative RT-PCR was performed using SYBR green mix (QIAGEN, Hilden, Germany) and Rotor-Gene Q (QIAGEN, Hilden, Germany). RT PCR primer sequences were the followings: *TBP*, 5’-AGCCAAGAGTGAAGAACAGTCC-3’ (forward) and 5’-CACAGCTCCCCACCATATTC-3’ (reverse); *HMOX1*, 5’-AACTTTCAGAAGGGCCAGGT-3’ (forward) and 5’-GTAGACAGGGGCGAAGACTG-3’ (reverse); *Superoxide dismutase 2* (*SOD2*), 5’-TCAATAAGGAACGGGGACAC-3’ (forward) and 5’-ACACATCAATCCCCAGCAGT-3’ (reverse); *E1A binding protein p30* (*EP300*),5’-AGGGGCAACAAGAAGAAACC-3’ (forward) and 5’-GAGGCGGATCACAAAGAAGA-3’ (reverse); *CREB Binding Protein* (*CREBBP*), 5’-CACTGCAGCCAGTGAAACC-3’ (forward) and 5’-CATGCTGGGCTTCTTCTTGT-3’ (reverse); *HDAC5*, 5’-AAGGTCCTCATCGTGGACTG-3’ (forward) and 5’-GCACAGAGGGGTCATTGTAGA-3’ (reverse). Amplification procedure was 95 °C for 5 min, followed by 40 cycles at 95 °C for 5 s, 60 °C for 10 s. Primers for *TBP* mRNA were used to normalize mRNA expression. All experiments were done in biological triplicate.

### Histone extraction

Histones were extracted from SH-SY5Y cells by acid extraction method in accordance with Shechter, et al.’s protocol [31]. Cells were washed with 1X PBS and pelleted by centrifugation at 1000 g for 3 min. Cell pellets were incubated in hypotonic lysis buffer (10 mM Tris, 1 mM KCl, 1 mM DTT, 1.5 mM MgCl_2_, 10 mM Sodium butyrate, 1 mM PMSF, and 7X protease inhibitor cocktail (Roche, Robert Rohrberg, Germany)) on a rotator at 4 °C for 30 min. The nuclei were collected by centrifugation at 10,000 x g at 4 °C for 10 min, and the pelleted nuclei were suspended in 0.4 N H_2_SO_4_ and placed on a rotator at 4 °C overnight. Next day, the nuclear lysate was collected from the supernatants after centrifugation at 14,620 x g at 4 °C for 10 min to remove nuclear debris. Histones were precipitated by TCA and washed with acetone-HCl and acetone. Precipitated histones were collected by centrifugation at 14,620 x g at 4 °C for 5 min, air-dried at room temperature for 15 min and then dissolved in distilled water. Histones were stored at -80 °C. All steps were carried out by keeping samples on ice whenever possible.

### Western blotting analysis

Isolated histones (2 or 3 μg per sample) were run on a 15 % PAGE. Histones were transferred to Nitrocellulose (BioRad Laboratories, Hercules, CA, USA) or PVDF (Millipore, Billerica, MA, USA) membranes. Membranes were blocked in TBS-T with 5 % skim milk (DifcoLaboratories, Detroit, Mich, USA) for 1 h at room temperature and incubated overnight at 4 °C with primary antibodies from Cell Signaling Technology, Danvers, MA, USA. The following antibodies were used: rabbit antibodies towards Histone H3 (1:1500), H3K9ac (1:1000) and H3K27ac (1:1000) and mouse antibodies to Histone H4 (1:1000). After washing, rabbit or mouse IgG-HRP secondary antibodies (Santa Cruz Biotechnology, Santa Cruz, CA, USA) were incubated for 1 h at room temperature and then developed at Pierce Enhanced chemiluminescence (ECL) kit (Thermo Scientific, Rockford, IL, USA). The level of acetylated histone was quantified by Image J. All experiments were done in biological triplicate.

### Statistical analysis

Mean ± S.E.M. of cell viability and mRNA expression levels were calculated by using Microsoft Excel 2010 (Microsoft, Redmond, WA, USA). One-way ANOVA analysis, followed by Duncan post hoc multiple comparisons, was performed using SPSS (ver. 20.0, SPSS Inc., Chicago, IL, USA). Statistical significance was set to *p* ≤ 0.05.

## Results

### MPP^+^-mediated changes of cell viability and *SOD2* and *HMOX1* expression in differentiated SH-SY5Y cells

**Fig. 1:**
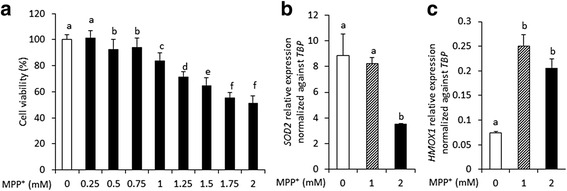
Changes of cell viability and mRNA expressions of *SOD2* and *HMOX1* in MPP^+^ treated cells. **a** MPP^+^ treatment decreased cell viability depending on concentrations of MPP^+^. **b** MPP^+^ treatment decreased mRNA expressions of *SOD2* related with clearing mitochondrial ROS. The results confirmed MPP^+^ induces apoptosis by way of inhibition of mitochondrial complex. **c** MPP^+^ treatment increased mRNA expressions of *HMOX1* related with the antioxidant pathway. The results consent *HMOX1* is induced by oxidative stress.; The data was indicated as the mean ± S.E.M. *P* < 0.05 was defined statistical significance. One-way ANOVA followed by Duncan post hoc test

We analyzed cell viability of SH-SY5Y cells upon MPP^+^ treatment using MTT assays. Treatment with MPP^+^ concentrations ranging from 0 to 2 mM decreased cell viability in a dose-dependent manner, down to about 52 % (Fig. 1a). To investigate whether MPP^+^ treatment changed the expression of genes related with the cellular anti-oxidative pathway in response to oxidative stress, mRNA expression levels of *SOD2* and *HMOX1* were analyzed (Fig. 1b and c). Gene expression level of *HMOX1* significantly increased at in 1 to 2 mM MPP^+^ treatment and *SOD2* significantly decreased at the 2 mM concentration. The results indicated that MPP^+^ indeed acted as a neurotoxicant in dopaminergic neuronal cells by inducing oxidative stress-mediated cell death. While the dose at 1 mM MPP^+^ is relatively low, it was sufficiently toxic to significantly elevate *HMOX1* gene expression and reduce cell viability. Consequently, for subsequent experiment we used the 1 mM dose to analyze the neuroprotective effects provided by PLE.

### Effects of PLE on MPP^+^-mediated cell death and *HMOX1* expression

**Fig. 2:**
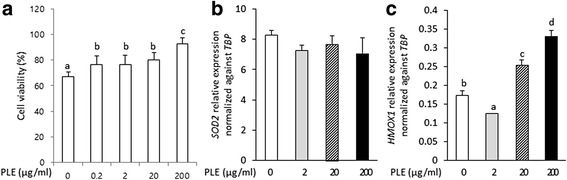
Cell viability and mRNA expressions of *SOD2* and *HMOX1* in PLE and MPP^+^ treated cells. **a** PLE treatment recovered 1 mM MPP^+^ treated cell viability depending on concentrations of PLE. The results suggested PLE has neuroprotective effects against MPP^+^-induced apoptosis. **b** PLE treatment didn’t alter mRNA expressions of *SOD2* in 1 mM MPP^+^ treated SH-SY5Y cells. **c** PLE treatment further activated mRNA expressions of *HMOX1* in 1 mM MPP^+^ treated SH-SY5Y cells. The results suggested PLE has neuroprotective effects on antioxidant function.; The data was indicated as the mean ± S.E.M. *P* < 0.05 was defined statistical significance. One-way ANOVA followed by Duncan post hoc test

To investigate the effect of PLE against MPP^+^-induced cell death, we pretreated SH-SY5Y cells with PLE in the range of 0 to 200 μg/ml for 4 h prior to a 24 h 1 mM MPP^+^ treatment. PLE pretreatment attenuated MPP^+^-induced cell death in a dose-dependent manner (Fig. 2a). Significantly, 200 μg/ml PLE recovered cell viability up to 93 % compared with MPP^+^ nontreated group. Interestingly, treatment with PLE did not alter *SOD2* gene expression levels, a key component of the antioxidant pathway (Fig. 2b), while *HMOX1* gene expression levels in 20-200 μg/ml PLE samples were significantly increased 1.5 to 1.9 fold, respectively, when compared to 0 μg/ml PLE group (Fig. 2c).

### Changes in acetylated levels of histones in MPP^+^ treated, differentiated SH-SY5Y cells

**Fig. 3:**
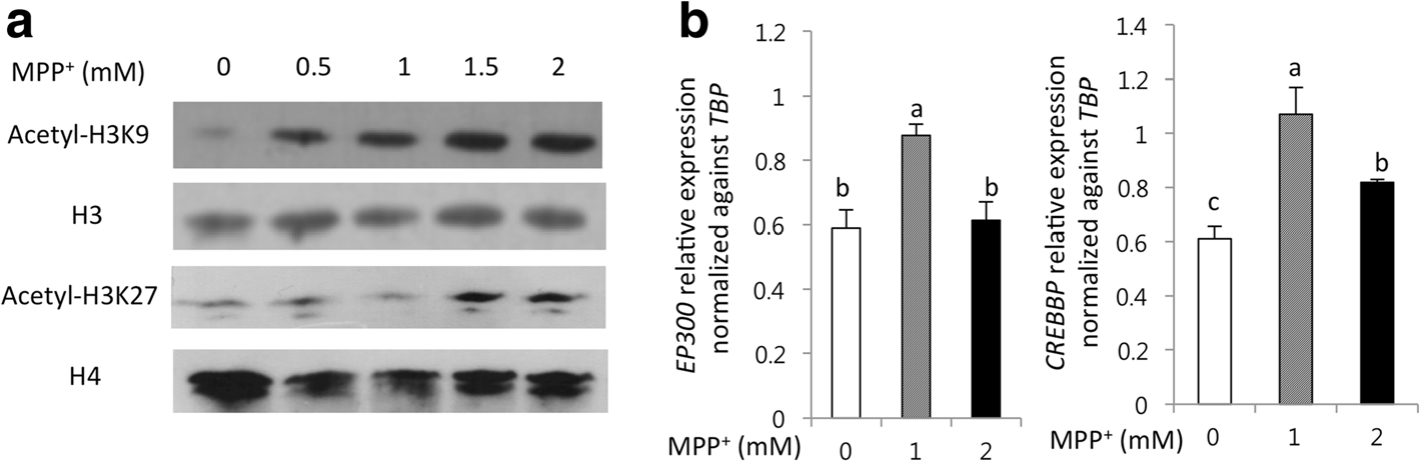
Acetylation of H3K9 and H3K27 and mRNA expressions of HATs in MPP^+^ treated cells. **a** MPP^+^ treatment increased acetylation levels of H3K9 and H3K27. **b** MPP^+^ treatment increased mRNA expressions of HATs such as *EP300* and *CREBBP*. Figure 3 suggested HATs increase acetylation levels of H3K9 and H3K27.; The data was indicated as the mean ± S.E.M. *P* < 0.05 was defined statistical significance. One-way ANOVA followed by Duncan post hoc test

To determine whether MPP^+^-induced cell death was a result of epigenetic changes, we investigated alteration in acetylation levels of histones and HAT gene expression levels. The acetylation levels of H3K9 and H3K27 (relative to total histones H3 and H4) were analyzed using western blot analysis. When treating cells with MPP^+^ ranging from 0.5 to 2 mM, we observed a linear correlation with increased acetylation levels of H3K9 and H3K27 (Fig. 3a). In addition, gene expression levels of the major HAT proteins, *EP300* and *CREBBP*, were also increased in the MPP^+^ treated samples (Fig. 3b). The results suggested that increased HAT expression under neurotoxic condition led to increased acetylation levels of H3K9 and H3K27.

### H3K9ac and H3K27ac and HDAC5 and HATs mRNA expressions in and MPP^+^ treated cells

**Fig. 4:**
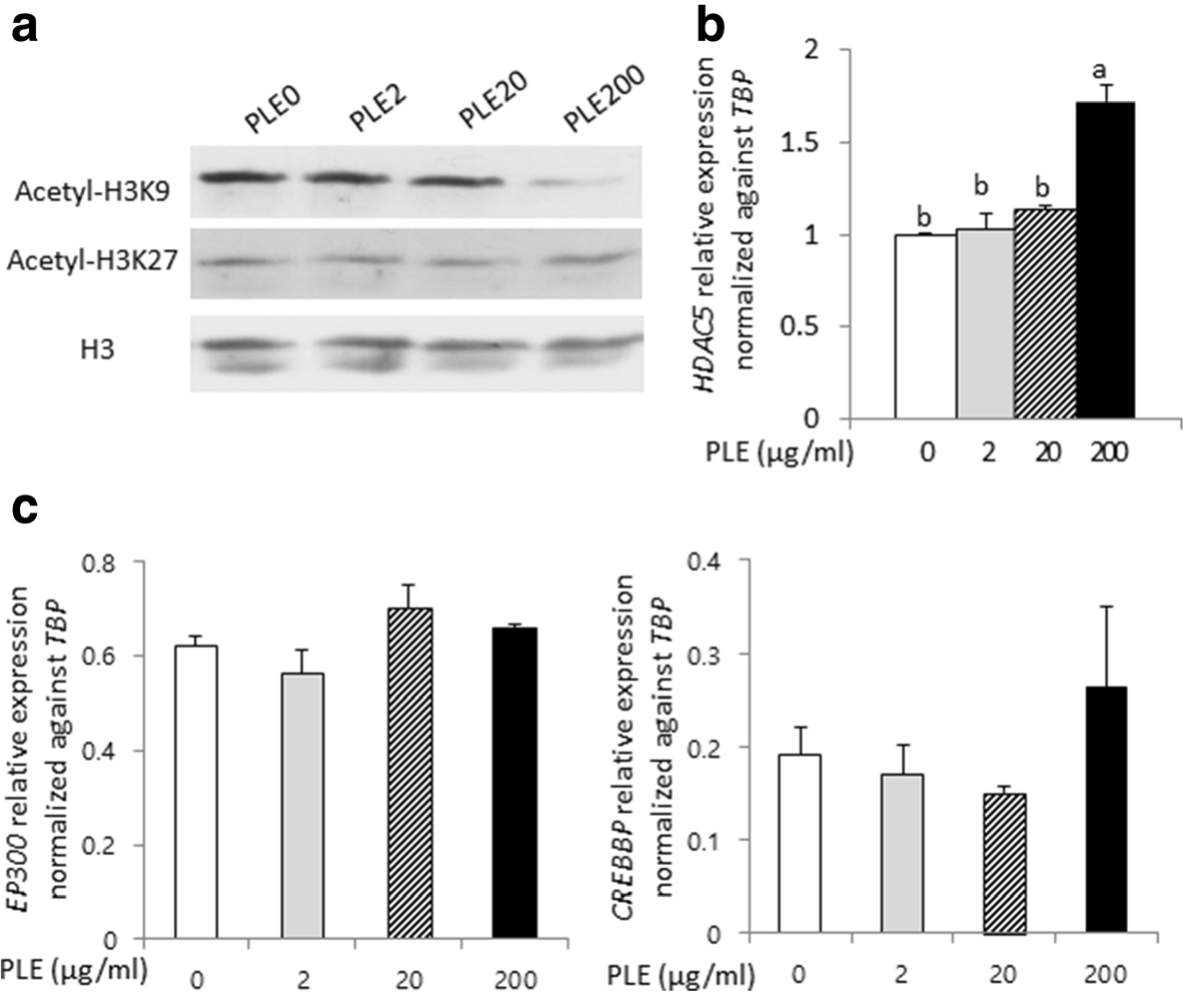
H3K9ac and H3K27ac and HDAC5 and HATs mRNA expressions in PLE and MPP^+^ treated cells. **a** 200 μg/ml PLE treatment decreased acetylation levels of H3K9 in 1 mM MPP^+^ treated cells. **b** PLE treatment did not significantly alter mRNA expressions of HATs in 1 mM MPP^+^ treated cells. **c** 200 μg/ml PLE treatment increased mRNA expressions of HDAC5 in 1 mM MPP^+^ treated cells. Figure 4 suggested HDAC5 rather than HATs decreases acetylation levels of H3K9. The data was indicated as the mean ± S.E.M. *P* < 0.05 was defined statistical significance. One-way ANOVA followed by Duncan post hoc test

Next, we determined whether pretreatment of PLE had neuroprotective effect against MPP^+^-induced cell death by means of epigenetic modulation. We investigated alteration of acetylation levels of histones and HATs and HDACs gene expression levels. We measured acetylation levels of H3K9 and H3K27 in MPP^+^ treated cells with and without PLE using western blotting analysis. In the PLE pretreated group, we observed that acetylation level of H3K9 decreased, but not H3K27 acetylation (Fig. 4a). The decreased acetylated H3K9 seemed to be due to induction of the *HDAC5* gene expression (Fig. 4b), rather than altered *EP300* and *CREBBP* expression (Fig. 4c), as *HDAC5* robustly increased with elevated PLE concentration. Our data suggest that *HDAC5* decreased acetylation levels of H3K9 in the 200 μg/ml PLE group, overriding the effect from HATs, potentiation PLE as an anti-neurotoxicant by tempering histone acetylation.

## Discussion

In this study, we investigated whether MPP^+^ induced cell death in the dopaminergic neuronal SH-SY5Y cells through epigenetic modulation, and whether PLE had a neuroprotective effect against cell death by reversing epigenetic changes. We demonstrated that MPP^+^-induced cell death decreased *SOD2* gene expression and increased the *HMOX1* gene expression levels. The results are consistent with previous reports showing downregulation of *SOD2* and upregulation of *HMOX1* under oxidative stress [15, 32, 33]. PLE was recently reported to have protective effect on neuronal cell death in studies both in vivo [6] and in vitro [7]. The positive influence of PLE on dopaminergic neuronal cells potentiates its use in attenuating the process of Parkinson’s disease [15, 16, 34]. However, the molecular mechanism of this protective effect remains unclear.

One possibility is that the PLE effect involves the antioxidant pathway. Our results demonstrated that PLE upregulated *HMOX1* gene expression, while it did not alter *SOD2* gene expression. This indicates that the PLE effect may be restricted to the HMOX1 pathway. *HMOX1* gene encodes heme oxygenase 1 (HMOX1), which catalyzes heme catabolism and plays a protective role as an antioxidant. *HMOX1* gene is activated in response to oxidative stress, mainly via a major transcriptional factor, *Nuclear factor erythroid 2 related factor 2* (*NRF2*), which exerts as a ROS scavenger [35]. A recent study showed that *Bromodomain protein 4* (*BRD4*) also transcriptionally activates *HMOX1* expression by targeting of SP1 binding sites in the *HMOX1* promoter [36]. *BRD4*, an acetylated histone-binding protein, is a key factor for regulation of gene expression [37, 38]. siRNA-mediated silencing of *Bromodomain protein 4* (*BRD4*), in the absence of stress, resulted in downregulation of *HMOX1* expression [36]. However, under oxidative stress, siRNA-mediated knockdown of *BRD4* upregulates *HMOX1* expression further via downregulation of *Kelch*-*like ECH*-*associated protein 1* (*KEAP1*), which inhibits the degradation of *NRF2* [36]. The obvious connection between hypoacetylated histone residues, especially H3K9, is reflected by the silencing of *BRD4* expression, since BRD4 binds to particularly acetylated histones.

The neurprotective effect of PLE on MPP^+^-induced cell death of the dopaminergic cells seems mediated by epigenetic modulation, supported by our observation of changes in acetylation of histone. Treatment of MPP^+^ increased gene expression levels of HATs such as *EP300* and *CREBBP*, resulting in hyperacetylation of H3K9 and H3K27. This is consistent with a previous report demonstrating that neurotoxicity induced hyperacetylation and transcriptional activation of pro-apoptotic genes, such as *PKC gamma* and *IL*-*8* [39]. Conversely, the pretreatment of PLE increased gene expression of *HDAC5*, resulting in hypoacetylation of H3K9. HDAC5 has been reported to involve neuronal cell survival, proliferation, and differentiation possibly via regulation of Myocyte enhancer factor-2 (MEF2) [40–42]. The resulting hypoacetylation upon PLE treatment may mimic the effect of BRD4 silencing under stress, leading to upregulated *HMOX1* expression.

Our study of the neuroprotective properties of PLE suggest that epigenetic modulation is a key component of neurotoxic stress, and that therapeutic intervention should consider this avenue of treatment in diseases such as Parkinson’s disease.

## Conclusions

MPP^+^-induced cell death in differentiated SH-SY5Y cells was significantly reduced by PLE pretreatment in a dose-dependent manner, accompanied by induced expression of *HMOX1*. MPP^+^-treatment increased the expression of HATs and consistently increased acetylation of H3K9 and H3K27, while PLE pretreatment attenuated the hyperacetylation in H3K9, coincided with increased expression of *HDAC5* without changes in HAT expression. Our study suggested that MPP^+^ treatment induced cell death in dopaminergic SH-SY5Y cells via upregulation of HATs and hyperacetylation of H3K9 and H3K27 and PLE pretreatment may attenuate the cell death, in part, by upregulation of *HMOX1* expression through hypoacetylation of H3K9, possibly mediated by HDAC5 upregulation. Further study is needed to identify direct targets for HDAC5-mediated hypoacetylation of H3K9 in the neuroprotective effects and to investigate whether it is recapitulated in vivo.

## Abbreviations

*BRD4*, *Bromodomain protein 4*; DMEM, Dulbecco’s modified Eagle’s medium; DMSO, dimethyl sulfoxide; *EP300*, *E1A binding protein p30*; FBS, fetal bovine serum; H3K27, histone H3 lysine 27; H3K27ac, acetylated lysine 27 of histone H3; H3K9, histone H3 lysine 9; H3K9ac, acetylated lysine 9 of histone H3; HAT, histone acetyltransferases; HDAC, histone deacetylases; *HMOX1*, *heme oxygenase 1*; MPP^+^, 1-methyl-4-phenylpyridinium; *NRF2*, *nuclear factor erythroid 2 related factor 2*; PBS, phosphate buffered saline; PLE, *Paeonia lactiflora* extract; ROS, reactive oxygen species; *SOD2*, *superoxide dismutase 2*
